# Fibroblast growth factor 2 contributes to the effect of salidroside on dendritic and synaptic plasticity after cerebral ischemia/reperfusion injury

**DOI:** 10.18632/aging.103308

**Published:** 2020-06-09

**Authors:** Sisi Li, Yechen Lu, Daofang Ding, Zhenzhen Ma, Xiangxin Xing, Xuyun Hua, Jianguang Xu

**Affiliations:** 1 School of Rehabilitation Science, Shanghai University of Traditional Chinese Medicine, Shanghai 201203, PR China; 2 Department of Rehabilitation Medicine, Yueyang Hospital of Integrated Traditional Chinese and Western Medicine, Shanghai University of Traditional Chinese Medicine, Shanghai 200437, PR China; 3 Department of Hand Surgery, Huashan Hospital, Fudan University, Shanghai 200040, PR China; 4 Department of Trauma and Orthopedics, Yueyang Hospital of Integrated Traditional Chinese and Western Medicine, Shanghai University of Traditional Chinese Medicine, Shanghai 200437, PR China

**Keywords:** salidroside, fibroblast growth factors, dendrite, synaptic plasticity, stroke

## Abstract

Ischemic stroke, a serious neurological disease, is associated with cell death, axonal and dendritic plasticity, and other activities. Anti-inflammatory, anti-apoptotic, promote dendritic and synaptic plasticity are critical therapeutic targets after ischemic stroke. Fibroblast growth factor-2 (FGF2), which is involved in the cyclic adenosine monophosphate (cAMP)/protein kinase A (PKA)/CAMP response element (CRE)-binding protein (CREB) pathway, has been shown to facilitate dendritic and synaptic plasticity. Salidroside (Sal) has been reported to have anti-inflammatory, anti-oxidative, and anti-apoptotic effects; however, the underlying mechanisms of Sal in promoting dendritic and synaptic plasticity remain unclear. Here, the anti-inflammatory, anti-apoptotic, dendritic and synaptic plasticity effects of Sal were investigated in vitro in PC12 cells under oxygen-glucose deprivation/reoxygenation (OGD/R) conditions and in vivo in rats with middle cerebral artery occlusion/reperfusion (MCAO/R). We investigated the role of Sal in promoting dendritic and synaptic plasticity in the ischemic penumbra and whether the FGF2-mediated cAMP/PKA/CREB pathway was involved in this process. The present study demonstrated that Sal could significantly inhibit inflammation and apoptosis, and promote dendritic and synaptic plasticity. Overall, our study suggests that Sal is an effective treatment for ischemic stroke that functions via the FGF2-mediated cAMP/PKA/CREB pathway to promote dendritic and synaptic plasticity.

## INTRODUCTION

Stroke is currently the second leading cause of death worldwide and can be classified into haemorrhagic and ischemic stroke. Among stroke patients, more than 80% suffer from cerebral ischemia, which is caused by the blockage of an artery supplying blood to the brain [1, 2].

Recent studies have shown that acute ischemic stroke leads to diverse pathophysiological changes, such as brain oedema, neuronal damage and synaptic dysfunction [3]. The behavioural changes and functional recovery after ischemic stroke are closely related to dendrite and synaptic plasticity [4]. Synapses are plastic, a phenomenon that is governed by the temporal patterns of presynaptic and postsynaptic activity. Postsynaptic activity can be determined by the properties of dendrites, indicating that dendrites play an important role in, and, to a certain extent, dominate synaptic plasticity [5]. While the initial degeneration of dendrites may not lead to the death of many injured neurons, when allowed to continue, dendrites will gradually degenerate, leading to a decrease in synaptic efficiency and, eventually, neuron death [6]. Therefore, available treatment interventions may be able to retard early dendritic degeneration to prevent the death of injured neurons.

First defined by Astrup J, the ischemic penumbra is a region characterized by extremely dynamic biochemical changes in the acute stage of cerebral ischemia that is not yet irreversibly impaired [7]. As an area of metabolically damaged tissue located around the most seriously affected ischemic centre, local cerebral blood flow can be restored by timely therapeutic intervention [8, 9].

It has been suggested that growth factors may be therapeutic targets for ischemic stroke [10]. Fibroblast growth factor-2 (FGF2, also known as bFGF) is a single-chain polypeptide containing 146 amino acids that serves as an important component of the FGF superfamily [11, 12]. The biological activity of FGF2 is mediated by its binding to a high-affinity cell surface receptor, FGF receptor 1 (FGFR1) [13]. FGF2 plays a critical role in cell–cell signaling between neurons during development and is thought to be responsible for neurogenesis, neuroprotection, and synaptic plasticity [14–16]. FGF2 enhances axonal branching and synaptogenesis in neurons and accelerates the bifurcation and growth of axonal branches [17]. FGF2 has been proposed to contribute to the recovery of neurologic function by increasing the dendritic arborization and spine density after ischemic brain injury [18]. By promoting axon spouting and new synapse formation, FGF2 can reduce the infarct size and promote the restoration of sensory motor functions.

FGF2 is involved in the cyclic adenosine monophosphate (cAMP)/protein kinase A (PKA) pathway, and FGF2 can be promoted by PKA to promote cell survival [19]. Studies have demonstrated that cAMP/PKA is involved in synaptic development and plasticity in the cortex [20]. It has been shown that cAMP is associated with synaptic neurotransmitters between motor and sensory neurons [21]. Physiologically, PKA acts as a central transducer in cAMP signaling, playing an essential role in a variety of physiological and developmental processes, such as learning and memory [22], neuron differentiation regulation, and especially, axonal/dendritic morphogenesis coordination [23]. In addition, the mechanism underlying the effects of cAMP/PKA on ischemia/reperfusion injury is correlated with the regulation of apoptosis [24] and inflammation [25]. CAMP response element (CRE)-binding protein (CREB) is the target of PKA, and the PKA**-**mediated phosphorylation of CREB at Ser133 is a well-characterized CREB activation mechanism. Interestingly, CREB is also a downstream molecule of FGF2/FGFR1 signaling [26, 27]. Numbers of studies have demonstrated that CREB acts as a transcription factor and plays a key role in promoting cell metabolism, proliferation, survival and remodelling of dendrites and axons [28, 29]. These findings demonstrate that the FGF2-mediated cAMP/PKA/CREB pathway has a neuroprotective effect by modulating the inflammatory response in ischemic brain injury.

**Figure 1:**
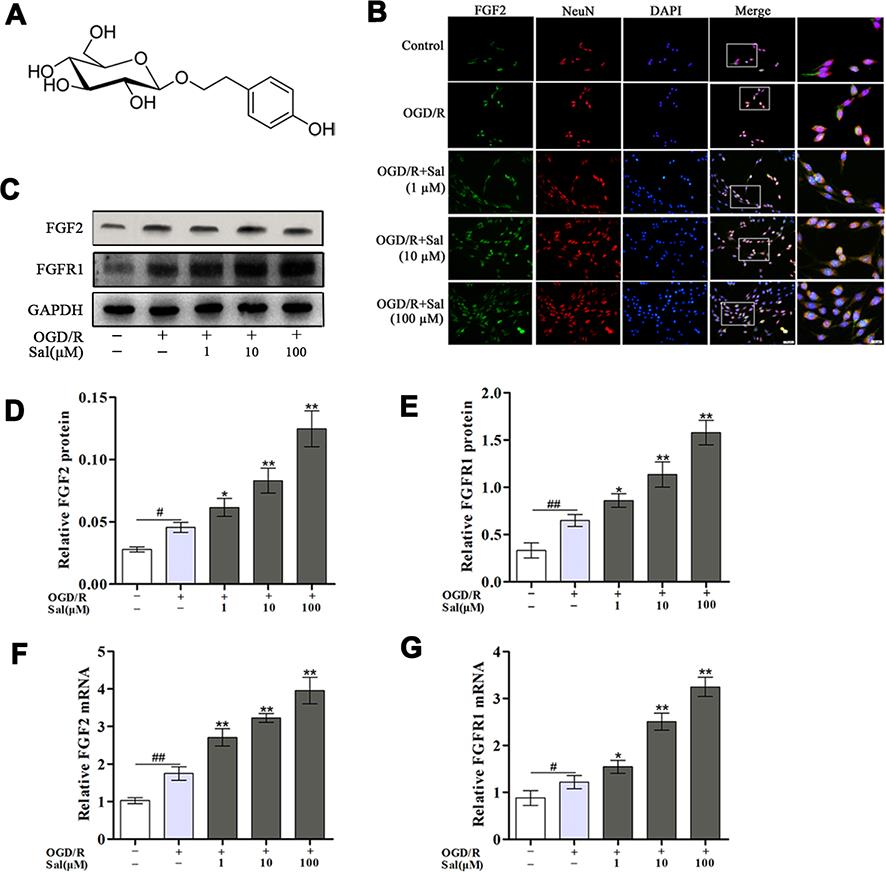
**Sal increases FGF2 and FGFR1 expression in PC12 cells after OGD/R.** (**A**) The chemical structure of Sal. (**B**) Double staining for FGF2-positive (green) and NeuN-positive (red) neurons (scale bars are 20 μm and 10 μm). (**C**–**E**) Representative western blot bands and protein expression of FGF2 and FGFR1 in PC12 cells. GAPDH was used as a protein loading control and for band density normalization. (**F**, **G**) The mRNA expression levels of FGF2 and FGFR1. Values are expressed as the mean ± SD. ^#^*p* < 0.05, ^##^*p* < 0.01 vs. control; ^*^p < 0.05, ^**^p < 0.01 vs. OGD/R.

Salidroside (Sal, Figure 1A shows the chemical structure of Sal), the major phenylpropanoid glycoside extract from *Rhodiola rosea* L, has diverse pharmacological activities. Many recent reports and reviews have highlighted that Sal may exert anti-inflammatory [30], neuroprotective effects [31] and improve cognitive function [32] both in vitro and in vivo. Previous studies have also indicated that Sal exhibits potential neuroprotective activity by regulating genes related to nerve synaptic plasticity [33, 34].

In the current study, we hypothesized that Sal may alleviate ischemia/reperfusion injury by reducing inflammation, inhibiting apoptosis and promoting dendritic and synaptic plasticity in the ischemic penumbra. Our study also investigated the role of FGF2**-**mediated cAMP/PKA/CREB pathway participates in the effect of Sal.

## RESULTS

### Sal upregulates FGF2/FGFR1 under OGD/R conditions in PC12 cells

Western blot, qPCR and immunofluorescence were performed to explore the effects of Sal on FGF2/FGFR1 mRNA and protein expression. The qPCR and western blot results suggested that OGD/R obviously elevated the mRNA and protein expression levels of FGF2/FGFR1, and the expression levels of FGF2/FGFR1 were higher in the Sal-pre-treated groups compared with the OGD/R group (Figure 1C–1G). The results of immunofluorescence staining for FGF2/NeuN were consistent with those of western blot and qPCR (Figure 1B). Those findings suggested that Sal efficiently increased the expression of FGF2/FGFR1.

### Sal attenuates OGD/R-induced proinflammatory cytokine secretion

**Figure 2:**
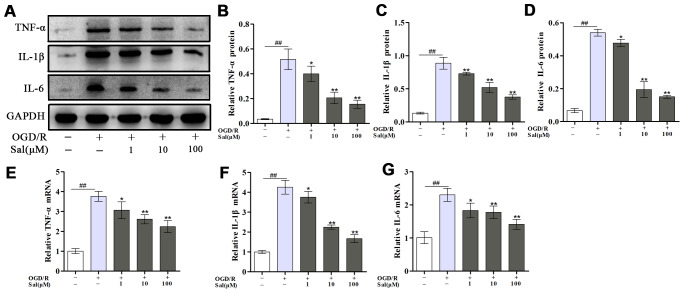
**Sal inhibits OGD/R-induced proinflammatory cytokine secretion.** (**A**–**D**) Optical density analysis of the TNF-α, IL-1β and IL-6 proteins. (**E**–**G**) The mRNA expression levels of TNF-α, IL-1β and IL-6. Values are expressed as the mean ± SD. ^#^*p* < 0.05, ^##^*p* < 0.01 vs. control; ^*^p < 0.05, ^**^p < 0.01 vs. OGD/R.

The effect of Sal on neuroinflammation induced by OGD/R was detected based on changes in inflammatory cytokine production. The results showed the protein and mRNA levels of proinflammatory mediators, including tumor necrosis factor alpha (TNF-α), interleukin-1β (IL-1β) and IL-6 were increased after OGD/R, Sal significantly reversed the inflammation induced by OGD/R (Figure 2).

### Sal inhibits neuronal apoptosis induced by OGD/R

**Figure 3:**
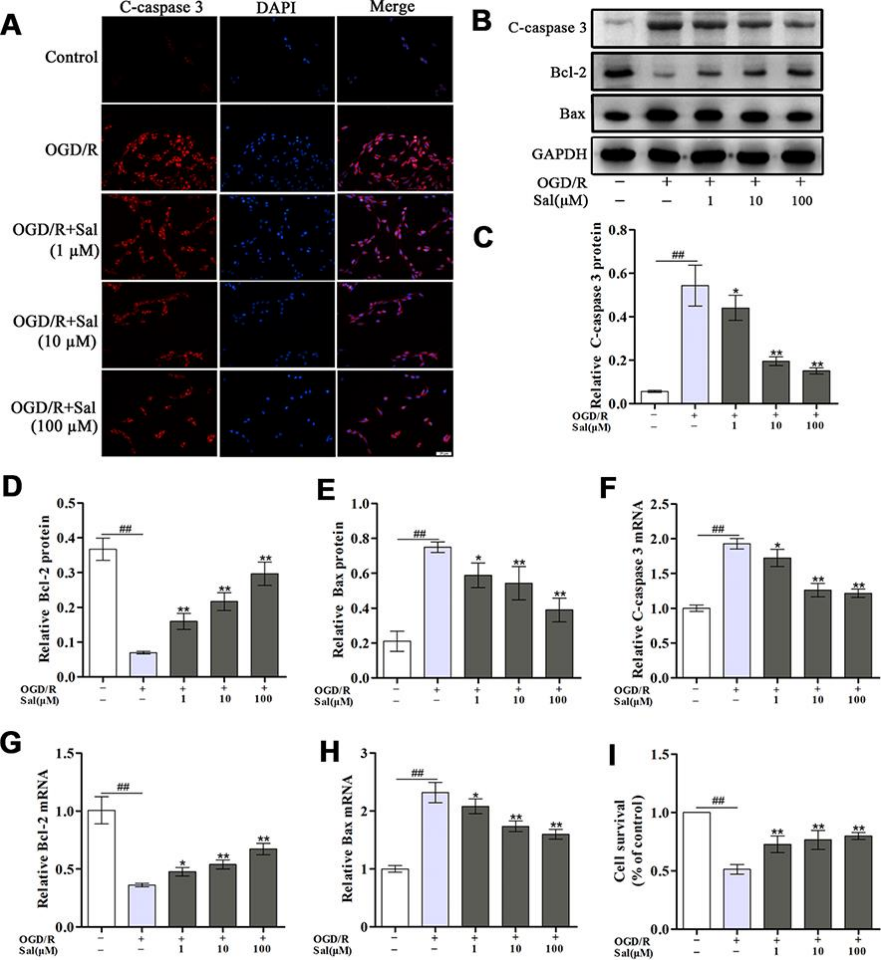
**Sal prevents OGD/R-induced neuronal apoptosis.** (**A**) Immunofluorescence staining of c-caspase 3 (the scale bar is 20 μm). (**B**–**E**) Representative western blot bands and protein expression of c-caspase 3, Bcl-2 and Bax in each group. (**F**–**H**) QPCR data showing the mRNA expression levels of c-caspase 3, Bcl-2 and Bax. (**I**) The CCK-8 assay was performed to assess cell proliferation. Values are expressed as the mean ± SD. ^#^*p* < 0.05, ^##^*p* < 0.01 vs. control; ^*^p < 0.05, ^**^p < 0.01 vs. OGD/R.

Immunofluorescence staining of cleaved caspase 3 (c-caspase 3) was performed to determine whether Sal could prevent neuronal apoptosis. As shown in Figure 3A, OGD/R increased neuronal apoptosis while the neurons in the Sal-pre-treated groups exhibited decreased c-caspase 3 staining. In addition, the expression levels of c-caspase 3, B-cell lymphoma-2 (Bcl-2) and Bcl-2-associated X protein (Bax) were analysed by western blot (Figure 3B-3E) and qPCR (Figure 3F–3H), the results showed that the incidence of apoptosis was significantly increased after OGD/R and decreased in Sal-pre-treated groups. The CCK-8 assay results indicated that compared with that in the OGD/R group, the cell viability in the Sal-pre-treatment groups was markedly increased (Figure 3I). These results indicated that Sal treatment significantly inhibited OGD/R-induced neuronal apoptosis.

### Sal promotes the production of synaptic-associated proteins after OGD/R

**Figure 4:**
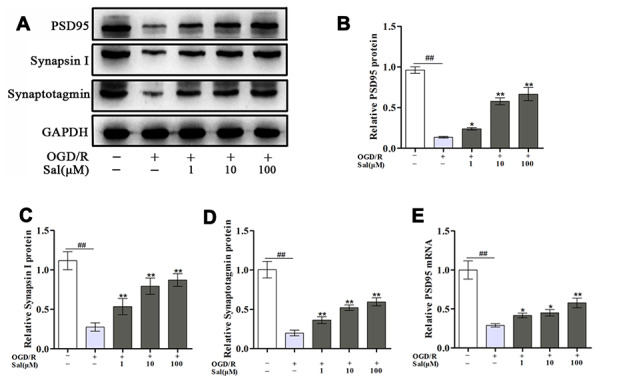
**Sal promotes the growth of synaptic-associated proteins after OGD/R.** (**A**–**D**) Protein expression and quantification analysis of PSD95, synapsin I and synaptotagmin in each group. (**E**) QPCR data for PSD95. Values are expressed as the mean ± SD. ^#^*p* < 0.05, ^##^*p* < 0.01 vs. control; ^*^p < 0.05, ^**^p < 0.01 vs. OGD/R.

To investigate the effect of Sal on synaptic-associated proteins after OGD/R in PC12 cells, we detected changes in post-synaptic density protein 95 (PSD95), synapsin I and synaptotagmin. Compared with those in the control group, the expression levels of PSD95, synapsin I and synaptotagmin under OGD/R conditions were markedly decreased. Significantly increased level of those were observed in the Sal-pre-treated groups compared to the OGD/R group (Figure 4), suggesting that Sal promoted the growth of synaptic-associated proteins after OGD/R.

### Sal reduces the brain infarct volume and neurological deficits in rats with MCAO/R

**Figure 5:**
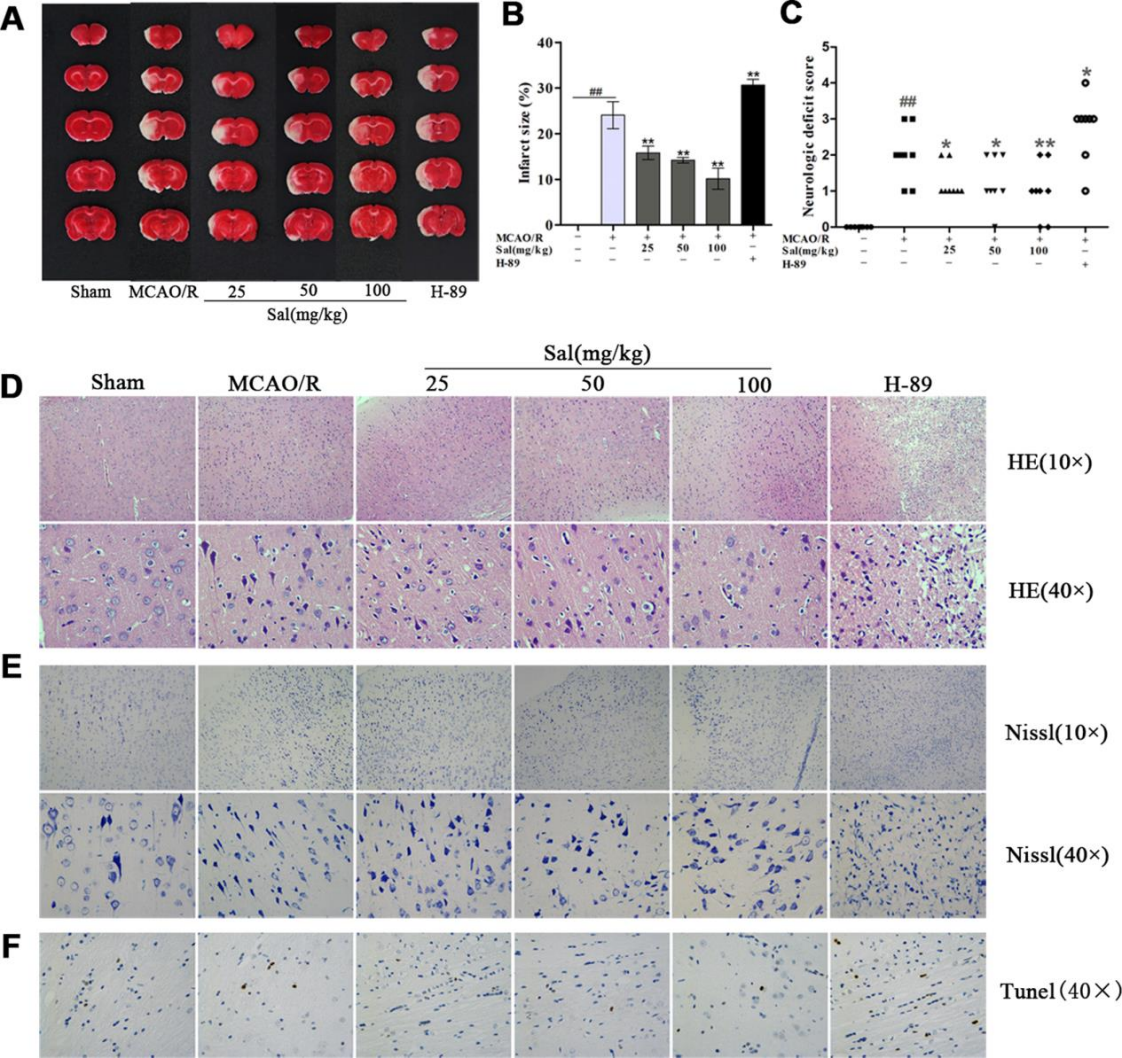
**Sal ameliorates tissue structure damage in the ischemic penumbra after MCAO/R.** (**A**, **B**) Representative images of ischemic lesions and statistical analysis of infarct volume at 7 days postinjury. (**C**) Neurological deficits. (**D**) HE staining at 7 days after MCAO/R (the scale bars are 100 μm and 20 μm). (**E**) Nissl staining images (the scale bars are 100 μm and 20 μm). (**F**) TUNEL staining in the ischemic penumbra (the scale bar is 20 μm). Values are expressed as the mean ± SD. ^#^*p* < 0.05, ^##^*p* < 0.01 vs. sham; ^*^p < 0.05, ^**^p < 0.01 vs. MCAO/R.

We investigated the effect of Sal and H-89 (inhibitor of PKA) on infarct size on day 7 after middle cerebral artery occlusion/reperfusion (MCAO/R). Infarct size was decreased after Sal treatment as determined by 2,3,5-triphenyltetrazolium chloride (TTC) staining. The H-89 group had larger infarct volumes than other groups (Figure 5A, 5B). In addition, Sal treatment significantly reduced neurological deficits (Figure 5C). The effects of Sal on neuronal morphology after MCAO/R were determined by haematoxylin-eosin (HE) staining (Figure 5D). The arrangement of brain tissue in the Sal groups was more regular than the untreated group, while brain tissue damage in the H-89 group was more severe than that in the other groups. In the H-89 group, brain tissue was irregularly arranged and unevenly stained, the intercellular space was increased, the number of cells was significantly decreased and the nuclei were small or absent. Nissl staining showed that the Sal groups had less neuronal apoptosis or necrosis than the MCAO/R group (Figure 5E). Furthermore, treatment with Sal resulted in a significant decrease in the number of apoptotic cells in comparison with that in the MCAO/R group, as identified by TUNEL staining, while the H-89 group exhibited increased apoptotic cell numbers compared with the MCAO/R group (Figure 5F).

### Sal upregulates the FGF2-mediated cAMP/ PKA/CREB signaling pathway in MCAO/R rats

**Figure 6:**
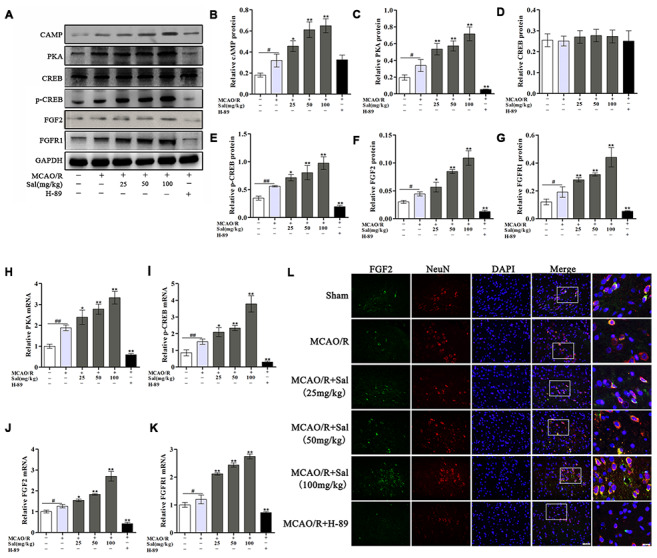
**Sal upregulates the FGF2-mediated cAMP/PKA/CREB signaling pathway following MCAO/R.** (**A**–**G**) Representative western blot bands of cAMP, PKA, CREB, p-CREB, FGF2 and FGFR1 in each group. (**H**–**K**) QPCR analysis of PKA, p-CREB, FGF2 and FGFR1 mRNA expression at 7 days after MCAO/R in different groups. (**I**) Double staining for FGF2-positive (green) and NeuN-positive neurons (red) neurons (the scale bars are 20 μm and 10 μm). Values are expressed as the mean ± SD. ^#^*p* < 0.05, ^##^*p* < 0.01 vs. sham; ^*^p < 0.05, ^**^p < 0.01 vs. MCAO/R.

We evaluated the expression of cAMP, PKA, CREB, p-CREB, FGF2 and FGFR1 in the ischemic penumbra by western blot and qPCR, revealing that the expression levels of cAMP, PKA, p-CREB, FGF2 and FGFR1 were markedly increased in the MCAO/R group compared with the sham group. The expression levels of FGF2 and FGFR1 were significantly increased after Sal treatment but were significantly decreased in the H-89 group compared with the MCAO/R group. There was no significant difference in cAMP expression between the MCAO/R and other groups (Figure 6A**-**6G). According to qPCR analysis, the mRNA expression levels of PKA, p-CREB, FGF2 and FGFR1 were significantly different between the MCAO/R and Sal groups (Figure 6H-6K). We also performed double-immunofluorescence staining for FGF2 and NeuN. As shown in Figure 6L, FGF2 was increased in the MCAO/R group compared with that in the same region in the sham group, while FGF2 in the Sal groups was expressed at substantially higher levels than that in the MCAO/R group.

### Sal treatment attenuates proinflammatory cytokine release after MCAO/R

**Figure 7:**
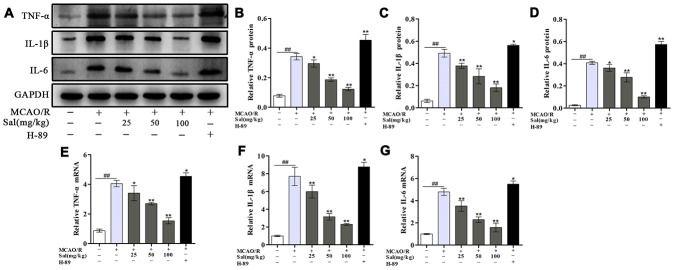
**Sal inhibits MCAO/R-induced inflammatory cytokine secretion.** (**A**–**D**) Optical density analysis of the TNF-α, IL-1β and IL-6 proteins. (**E**–**G**) QPCR results of TNF-α, IL-1β and IL-6 expression. Values are expressed as the mean ± SD. ^#^*p* < 0.05, ^##^*p* < 0.01 vs. sham; ^*^p < 0.05, ^**^p < 0.01 vs. MCAO/R.

The effect of Sal on neuroinflammation induced by MCAO/R was detected through changes in proinflammatory cytokine production. The data showed that the proinflammatory cytokines TNF-α, IL-1β and IL-6 were significantly elevated in the MCAO/R group, treatment with Sal significantly suppressed the amount of TNF-α, IL-1β and IL-6 (Figure 7).

### Treatment with Sal inhibits MCAO/R-induced neuron apoptosis

**Figure 8:**
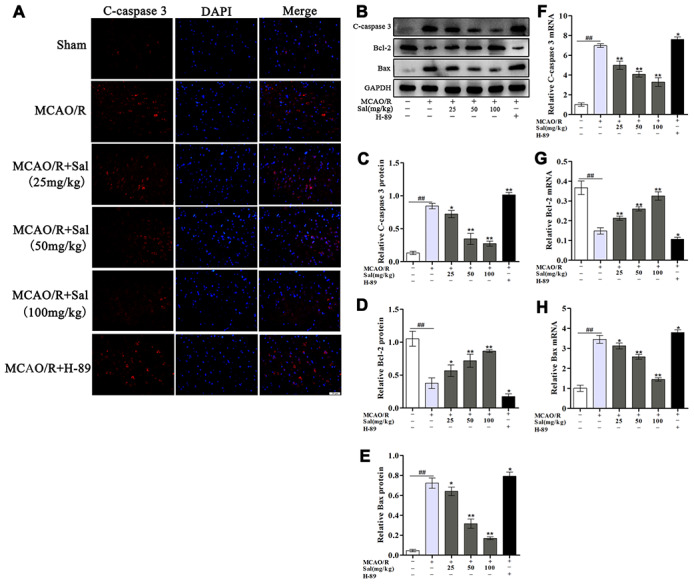
**Sal attenuates neuronal apoptosis after MCAO/R.** (**A**) Immunofluorescence staining of c-caspase 3 in sections from the ischemic penumbra in each group on day 7 post-MCAO/R (the scale bar is 20 μm). (**B**–**E**) Protein expression of c-caspase 3, Bcl-2 and Bax from the ischemic penumbra. (**F**–**H**) QPCR results of c-caspase 3, Bcl-2 and Bax expression. Values are expressed as the mean ± SD. ^#^*p* < 0.05, ^##^*p* < 0.01 vs. sham; ^*^p < 0.05, ^**^p < 0.01 vs. MCAO/R.

To assess whether Sal prevents neuronal apoptosis, we analysed c-caspase 3, Bcl-2 and Bax levels by western blot (Figure 8B**-**8E) and qPCR (Figure 8F**-**8H). Our results indicated that Sal treatment exhibited decreased neuronal apoptosis compared with MCAO/R group. Neuronal apoptosis in the H-89 group was significantly worse than that in the MCAO/R group. We also performed immunofluorescence staining for c-caspase 3, the results were consistent with western blot and qPCR analysis (Figure 8A). These results demonstrated that treatment with Sal might be an effective strategy for inhibiting apoptosis and protecting neurons through the FGF2**-**mediated cAMP/PKA/CREB signaling pathway.

### Sal promotes dendritic growth by upregulating the FGF2-mediated cAMP/PKA/CREB signaling pathway

**Figure 9:**
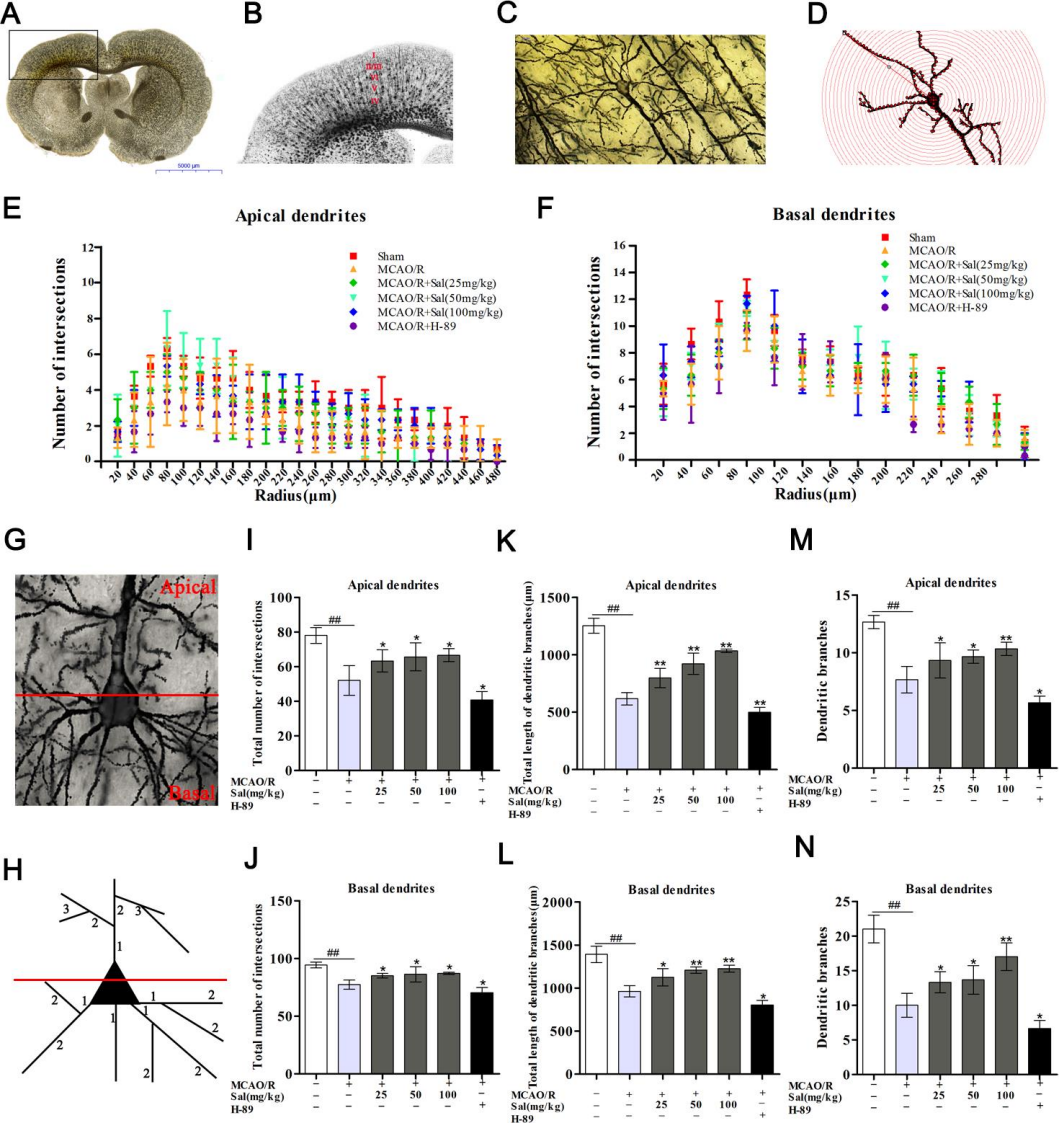
**Sal promotes dendritic growth by upregulating the FGF2-mediated cAMP/PKA/CREB signaling pathway.** (**A**, **B**) Representative coronal sections processed by Golgi staining methods after MCAO/R. (**C**, **D**) Example of a layer V pyramidal neuron (the scale bar is 20 μm). (**G**) Illustration of the demarcation between the apical (upper) and basal (lower) dendrites. (H) Dendritic segments are numbered in the proximal to distal direction from the soma. (**E**, **F**) Distribution of the dendritic intersections at an increasing distance from the soma. (**I**, **J**) Total number of intersections in each group. (**K**, **L**) Total length of dendritic branches. (**M**, **N**) Dendritic branches. Values are expressed as the mean ± SD. ^#^*p* < 0.05, ^##^*p* < 0.01 vs. sham; ^*^p < 0.05, ^**^p < 0.01 vs. MCAO/R.

Golgi-Cox staining clearly illustrated a significant decrease in the total number of intersections in both apical and basal dendrites after ischemic reperfusion injury. The number of intersections in apical and basal dendrites were significantly increased in the Sal groups compared with the MCAO/R group (Figure 9I, 9J). Additionally, we detected the total dendritic length, and the numbers of both apical and basal dendritic branches of layer V neurons in the penumbra. The total lengths of both apical and basal dendrites were significantly decreased after MCAO/R. Compared with the MCAO/R group, the total lengths of both apical and basal dendrites were increased in the Sal groups (Figure 9K, 9L). The total branches of both apical and basal dendrites in the Sal groups were significantly increased compared with those in the MCAO/R group (Figure 9M, 9N). While there were fewer dendrite intersections, fewer dendritic branches and shorter total dendrite length in the H-89 group.

### Sal promotes dendritic spine density and synaptic plasticity via upregulation of the FGF2-mediated cAMP/PKA/CREB signaling pathway

**Figure 10:**
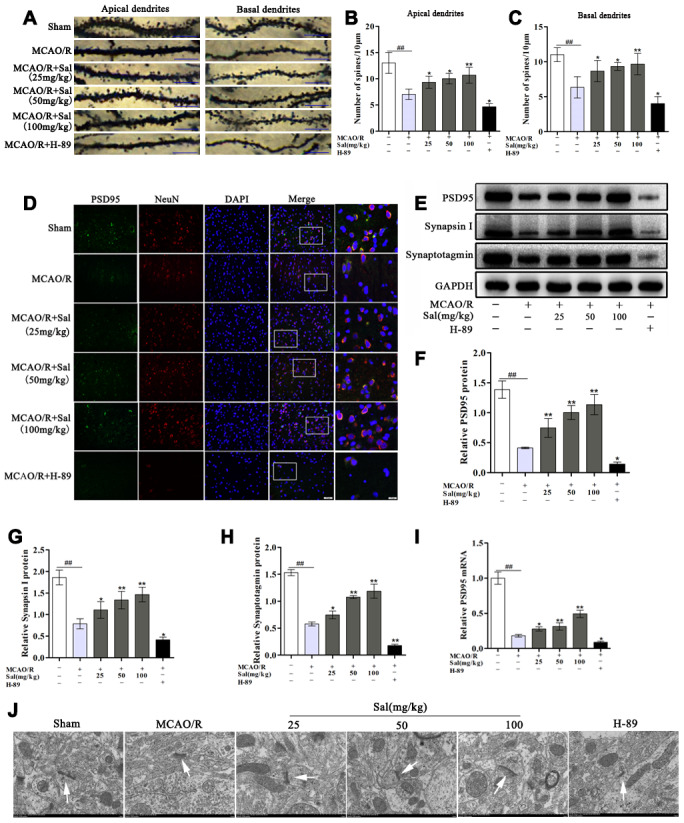
**Sal promotes increases in dendritic spine density and synaptic-associated protein expression via upregulating the FGF2-mediated cAMP/PKA/CREB signaling pathway.** (**A**) Examples of dendritic spines (the scale bar is 10 μm). (**B**, **C**) Density of dendritic spines. (**D**) Double immunofluorescence staining of PSD95-positive (green) and NeuN-positive (red) neurons from sections in each group on day 7 after MCAO/R (the scale bars are 20 and 10 μm). (**E**–**H**) Representative western blot bands of PSD95, synapsin I and synaptotagmin in each group. (**I**) QPCR data for PSD95. (**J**) Transmission electron microscopy showed the synaptic structures (the scale bar is 1 μm). Values are expressed as the mean ± SD. ^#^*p* < 0.05, ^##^*p* < 0.01 vs. sham; ^*^p < 0.05, ^**^p < 0.01 vs. MCAO/R.

To determine whether Sal attenuates dendritic spine damage, we detected changes in dendritic spine density. Both the apical and basal dendritic spine density in the MCAO/R group were markedly decreased compared with the sham group. And both the apical and basal dendritic spine density in the Sal groups were significantly increased compared with the MCAO/R group (Figure 10A–10C). To investigate the role of Sal in synaptic plasticity, we detected changes in synaptic proteins, including PSD95, synapsin I and synaptotagmin. The expression levels of PSD95, synapsin I and synaptotagmin were significantly decreased in the MCAO/R group compared with the sham group. Sal treatment reversed the expression of PSD95, synapsin I and synaptotagmin (Figure 10E**-**10I), while their expression in the H-89 group was remarkably decreased. The results of immunofluorescence staining for PSD95 were consistent with those of western blot and qPCR (Figure 10D). Furthermore, we examined the effect of Sal on synapse morphology in neurons. The electron microscopy results revealed that the sham group exhibited complete synaptic structures in normal neurons. The MCAO/R group exhibited damaged synaptic structures, and the number of synaptic vesicles was reduced in the ischemic penumbra neurons compared with those in the sham group. A remarkably thickness synaptic membrane, tighter synaptic connections, and more synaptic vesicles were observed in the Sal treatment groups compared with the MCAO/R group. The H-89 group exhibited more significant synapse morphology deficits than the MCAO/R group (Figure 10J).

## DISCUSSION

Previous studies have concentrated on the inhibition of neuroinflammation as a potential strategy for the treatment of stroke. However, the roles of dendritic modification and synaptic plasticity in neurons and their underlying molecular mechanisms remain unclear. Our results demonstrate that the FGF2**-**mediated cAMP/PKA/CREB plays a crucial role in dendritic modification and synaptic plasticity. In addition, Sal acts as an effective treatment option through the FGF2**-**mediated cAMP/PKA/CREB signaling pathway to reduce inflammation, apoptosis and promote dendritic and synaptic plasticity after ischemic stroke.

Ischemic stroke triggers the release of proinflammatory mediators, cell death, axonal damage, and regeneration inhibition. Anti-inflammation, anti-apoptosis and maintaining the survival of injured neurons is an important process for the protection and regeneration of the nervous system. Cerebral ischemia is followed by an inflammatory reaction, the release of various proinflammatory cytokines, including IL-6, IL-1β and TNF-α, is the main mechanism underlying ischemic inflammatory injury [35, 36]. TNF-α is a potent proinflammatory cytokine that exerts pleiotropic functions in ischemic brain injury [37]. IL-1β is a main mediator of inflammation and can induce neuronal apoptosis and promote the production of chemokines [38]. IL-6 plays a comprehensive role in cerebral ischemia and is most prominently identified in neurons of peri-ischemic regions [39]. In the current investigation, Sal was capable of inhibiting inflammation and apoptosis both in vivo and in vitro. It had a significant therapeutic effect on decreasing neurological deficits and alleviating the cerebral infarction volume induced by MCAO/R. And the numbers and morphous of neurons in the ischemic penumbra were better in the Sal treatment groups than in the MCAO/R group.

Synapses are the contact sites between neurons in the central nervous system and therefore contribute to the processing, transfer and storage of information. Dendritic spines are tiny protrusions scattered along the dendrites of many types of neurons and represent the major target of excitatory synapses [40]. Previous studies have shown that a loss of blood supply to the brain, such as a 90% reduction in blood flow, results in dendritic spine loss and irreversible dendritic damage within 10-20 min [41]. In the acute MCAO model, ischemic stroke not only induces neuronal death in the ischemic core area of the infarct but also damages the structure and neurons in the areas surrounding the core (the ischemic penumbra) [42]. The patterns of activity that induce synaptic plasticity at excitatory synapses are disrupted in the zone surrounding the infarct and in areas far from the infarct connected to the infarct area. Spines and dendrites of central neurons represent important sites of synaptic signaling and are the most vulnerable structures after a sudden disruption of blood flow. These changes have been shown to disrupt the neuronal circuitry and impair the function of brain synaptogenesis, and dendritic growth is necessary following cerebral stroke [43]. The dendrite length represents the total synapse space, and the spine density reflects the density of excitatory synapses to some extent. To increase synaptogenesis, dendritic arborization and increased spine density are potential morphological strategies that enable the brain to reorganize its neuronal circuits.

Recent studies have shown that FGF2 is increased and plays an indispensable role in recovery after ischemic insult [44, 45]. FGF2 is crucial for neurologic recovery by increasing dendritic length and spine density after ischemic brain injury. The FGF2**-**mediated aberrantly activated cAMP/PKA/CREB pathway is a critical immunological signaling cascade associated with dendritic and synaptic plasticity and thus contributes to recovery after ischemic stroke. The expression of cAMP, PKA and p-CREB was increased at 7 days after ischemic infarction [46, 47]. This pathway can stimulate axon and dendritic sprouting, elongation and branching; increase neurotransmitter release related to synaptic modulation; and strengthen signal transduction in synapses. Synaptotagmin is crucial for the docking of synaptic vesicles and fusion with neuron membrane [48]. Synapsin I, a protein involved in synaptic vesicle formation, synaptogenesis, and regulation of neurotransmitter release [49]. PSD-95 is a major scaffolding protein in the postsynaptic densities of dendritic spines [50]. A remarkable increase of synaptotagmin, synapsin I and PSD95 expression was revealed indicating the potential function of Sal in synaptogenesis. Golgi staining showed dendrite arbor atrophy and decreased spine density in the MCAO/R group. Compared with the MCAO/R, the H-89 group exhibited a significantly shorter total dendritic length, decreased dendritic complexity and loss of accompanying neurons. Sal improved the recovery of neurological function by increasing spine formation, dendritic elongation and branching, and the effects of high-dose Sal after stroke were more obvious.

The major limitations of this study should be noted. First, the study aimed mainly to evaluate the effects of Sal on dendritic and synaptic plasticity and the role of FGF2 in these effects. We did not use transgenic knockout or overexpression approaches to elucidate the biological role of FGF2. Second, Long-term potentiation and depression (LTP and LTD) were not used to detect changes at the functional level due to the experimental conditions. Thus, we should improve the experimental design in future studies.

**Figure 11:**
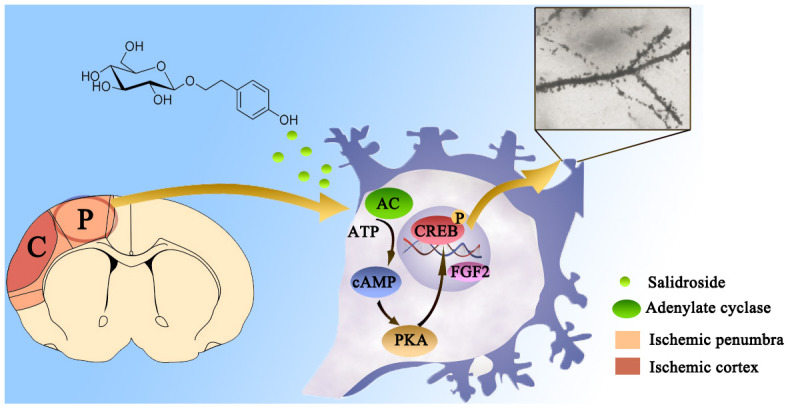
**Diagram of the FGF2-mediated cAMP/PKA/CREB signaling pathway contribution to the dendritic and synaptic plasticity of salidroside after focal cerebral ischemia/reperfusion injury.**

In conclusion, this study indicated that the intervention of Sal reduced inflammation and apoptosis, improved neuronal survival and enhanced dendritic and synaptic plasticity via the FGF2**-**mediated cAMP/PKA/CREB pathway (Figure 11). Our results demonstrated that Sal may be useful as a therapeutic agent for treating ischemic stroke.

## MATERIALS AND METHODS

### Reagents and antibodies

Rat adrenal pheochromocytoma cell lines (PC12 cells) were obtained from the Shanghai Institute of Cell Biology (Shanghai, China). Fetal bovine serum (FBS), penicillin/streptomycin, and Dulbecco’s modified Eagle’s medium (DMEM) were purchased from Gibco Life Technologies (Rockville, MD, US). Cell counting kit-8 (CCK-8) was purchased from Dojindo Laboratories (Tokyo, Japan). The In Situ Cell Death Detection Kit was purchased from Roche Applied Science (Indianapolis, IN, USA). Sal (C14H20O7, CAS#: 10338–51–9, purity > 98%) was purchased from Nanjing Zelang Medical Technological Co. Ltd (Nanjing, China). H-89 dihydrochloride (inhibitor of PKA, HY-15979A) was purchased from Med Chem Express (MCE, New Jersey, USA). Specific antibodies against synapsin I (ab8) and PSD95 (ab18258) were purchased from Abcam (Cambridge Science Park, Cambridge, UK). Specific antibodies against cAMP (DF7741), PKA (AF5450), CREB (AF6188), phosphorylated (p)-CREB (AF3189), FGF2 (DF6038), FGFR1 (AF6156), TNF-α (AF7014), IL-1β (AF5103), IL-6 (DF6087), c-caspase 3 (BF0711), Bcl-2 (AF6139), Bax (AF0120), synaptotagmin (AF6224) and NeuN (DF6145) were obtained from Affinity Biosciences (Cincinnati, OH, USA). Another NeuN antibody (MAB377) was purchased from Millipore (Billerica, MA, USA).

### Cell culture and oxygen-glucose deprivation/ reoxygenation (OGD/R)

PC12 cells were seeded onto 6-well plates in a humidified atmosphere at 37 °C with 5% CO_2_. PC12 cells were pre-treated with different concentrations of Sal for 24 h before OGD. Subsequently, the culture medium was replaced with DMEM without glucose, and the cells were subjected to hypoxic conditions with 5% CO_2_, 94% N_2_ and 1% O_2_ at 37 °C for 4 h. Then, the cells were fed normal culture medium and returned to the incubator under normoxic conditions for an additional 24 h. The viability of PC12 cells treated with different doses of Sal (1, 10 and 100 μM) was assessed by the CCK-8 assay.

### Animals

Sprague-Dawley male rats (weighing 250-280 g) were obtained from Shanghai Laboratory Animal Center (Shanghai, China). All animal care and experimental procedures were approved by the Animal Research Committee of Shanghai University of Traditional Chinese Medicine and were performed according to the Guide for the Care and Use of Laboratory Animals of the National Institutes of Health Guide. All animals were housed in standard plastic cages, maintained in a temperature-controlled environment (23-25 °C) under a 12-h light/dark cycle, and allowed free access to food and water. Animals were randomly divided into six groups (n=15 for each group): sham, MCAO/R+vehicle, MCAO/R+Sal (25 mg/kg), MCAO/R+Sal (50 mg/kg), MCAO/R+Sal (100 mg/kg) and MCAO/R+H-89.

### MCAO model, drug injection, and evaluation of neurological deficits

Rats were anesthetised with 2% sodium pentobarbital (30 mg/kg, i.p.). Briefly, the common carotid artery (CCA) and external carotid artery (ECA) were exposed through a ventral cervical midline incision. Microvascular clips were temporarily placed on the CCA and internal carotid artery (ICA). A fine silicon-coated surgical nylon monofilament thread (0.36 ± 0.02 mm; L3600, Jia Ling Biotechnology Co. Ltd., Guangzhou, China) was inserted from the CCA bifurcation along the ICA at approximately 18 mm to block the origin of the left MCA. Reperfusion was initiated by gently withdrawing the nylon monofilament thread after 2 h of occlusion. Rats in the sham group were operated on in the same manner except that the surgical nylon monofilament thread. Saline or different concentrations of drugs were immediately injected at the beginning of ischemia (0 h) and after reperfusion (1 h) and then once per day for seven consecutive days. The dosages of H-89 used in this study were based on those in previously published studies [51, 52].

The neurological deficits of animals were evaluated on day 7 after MCAO. Neurological function was determined using a 4-point evaluation as described previously [53]: 0 = no observable neurological deficit; 1 = failure to fully extend the right forepaw; 2 = circling to the right; 3 = failing to the right side; and 4 = no spontaneous ambulation and/or complete immobility.

### Cerebral infarct assessment

Rat brains were quickly removed and coronally sectioned to a thickness of 2 mm. Subsequently, the sections were quickly immersed in a solution containing 2% TTC at 37°C in the dark for 20 min and fixed in fresh 4% paraformaldehyde in phosphate-buffered saline (PBS) for at least 1 h.

### Tissue preparation and HE, Nissl, and TUNEL staining

On day 7, under deep anaesthesia with 2% sodium pentobarbital, rats were perfused through the ascending aorta with 200 ml of saline (0.9% NaCl), followed by 250 ml of ice cold fresh 4% paraformaldehyde in 0.1 M PBS (pH 7.4). After the brains containing the ischemic penumbra were fixed, paraffin and serial sections (5 μm thick) were mounted on slides coated with poly-L-lysine for HE, Nissl, TUNEL staining and immunofluorescence analyses. For histological staining, sections were stained with haematoxylin and eosin for HE staining and cresyl violet for Nissl staining, respectively. Apoptosis was assessed histologically and with an in situ cell death detection kit, according to the manufacturer’s instructions. An Olympus microscope was used to collect images.

### Immunofluorescence

Prepared sections were deparaffinized with xylene (changed twice, 10 min each) and rehydrated in a graded ethanol series. After rinsing in PBS three times, the sections were treated with 0.01 M sodium citrate buffer at 95 °C for 20 min for antigen retrieval, followed by incubation with 3% H_2_O_2_ for 10 min. Slices were blocked in 10% normal goat serum with 0.3% Triton-X-100 in 0.01 M PBS at room temperature for 1 h. Then, the slides were incubated with the primary antibody (FGF2, 1:200; c-caspase 3, 1:200; PSD95, 1:200; NeuN, 1:200) overnight at 4 °C. After incubation with the primary antibody, the sections were probed at 37 °C for 30 min with the following labelled secondary antibodies: DyLight 594-labelled goat anti-mouse IgG (1:200) and fluorescein isothiocyanate (FITC)-conjugated AffiniPure goat anti-rabbit IgG (1:200). Nuclei were stained with 4,6-diamidino-2-phenylindole (DAPI) for 5 min. Immunofluorescence was examined in the ischemic penumbra region of the cerebral cortex.

### Western blot analysis

Cell supernatants and tissues were collected for protein assays. The extracted proteins were first quantified by the BCA protein assay. Cellular samples containing 30 μg of protein and tissue samples containing 80 μg of protein were separated on SDS/PAGE gels and transferred onto PVDF membranes. Then, the membranes were blocked for 1 h at room temperature (RT) with 5% skim milk and incubated with the appropriate primary antibodies at 4°C overnight. The concentrations of the primary antibodies were as follows: cAMP (1:1000), PKA(1:500), CREB (1:500), p-CREB (1:500), FGF2 (1:500), FGFR1 (1:500), TNF-α (1:500), IL-1β (1:500), IL-6 (1:500), Bax (1:500), Bcl-2 (1:500), c-caspase 3 (1:500), PSD95 (1:300), synaptotagmin (1:500), synapsin I (1:1000) and GAPDH (1:1000). The membranes were rinsed three times with TBST and incubated with horseradish peroxidase-conjugated secondary antibodies at room temperature for 2 h. The protein bands were visualized using an enhanced chemiluminescence (ECL) system (Beyotime Corp, China). Quantitative densitometric analysis was performed using AlphaEaseFC (version 4.0).

### Quantitative real-time PCR

Total RNA was extracted from cultured cells and tissue samples using TRIzol reagent (Invitrogen, USA), and 1 μg of total RNA from each sample was used to synthesize cDNA (A3500, Promega). Real-time PCR amplification was performed on a Light Cycler480 system (Roche, USA) using SYBR Green (QPK-212, Tokyo, Japan). The cycling conditions were as follows: 95 °C for 5 min, followed by 40 cycles of 95 °C for 10 sec, 60 °C for 10 sec, and 72 °C for 10 sec; each sample was tested in triplicate. Tubulin was selected as an internal reference, and the gene expression levels were calculated using the 2^-ΔΔCt^ method. The primers for PKA, p-CREB, FGF2, FGR1, TNF-α, IL-1β, IL-6, c-caspase 3, Bcl-2, Bax and PSD95 are listed in Table 1.

**Table 1:** Primers used for real-time PCR analysis.

**Genes**	**Forward primers**	**Reverse primers**
PKA	GGACAAGCAGAAGGTGGTGAAGC	ACCAGGCACGTACTCCATGACC
p-CREB	TGTTGTTCAAGCTGCCTCTGGTG	GCTTCTTCAGCAGGCTGTGTAGG
FGF2	GGTGGAAGGCTGGTCGTTGTG	TCCAGGAGACTGCCGTGACG
FGFR1	CTCTGCATGGTTGACCGTTCTGG	GCTCTTCTTGGTGCCGCTCTTC
TNF-α	GCATGATCCGAGATGTGGAACTGG	CGCCACGAGCAGGAATGAGAAG
IL-1β	ATCTCACAGCAGCATCTCGACAAG	CACACTAGCAGGTCGTCATCATCC
IL-6	AGGAGTGGCTAAGGACCAAGACC	TGCCGAGTAGACCTCATAGTGACC
C-caspase 3	GTACAGAGCTGGACTGCGGTATTG	AGTCGGCCTCCACTGGTATCTTC
Bcl-2	ACGGTGGTGGAGGAACTCTTCAG	GGTGTGCAGATGCCGGTTCAG
Bax	CCAGGACGCATCCACCAAGAAG	GCTGCCACACGGAAGAAGACC
PSD95	TCCAGTCTGTGCGAGAGGTAGC	GGACGGATGAAGATGGCGATGG
Tubulin	ATGCCAACCTTGAAGCCAGTG	GCTTTGAGCCAGCCAACCAGA

### Transmission electron microscopy

Tissue samples were obtained from the ischemic stroke penumbra region without heart perfusion and kept in a solution containing 2.5 % glutaraldehyde overnight. After washing in PBS three times, the samples were fixed in 1% osmic acid for 1 h and stained with 1% uranyl acetate for 2 h. After routine gradient dehydration with an acetone solution, tissues were embedded for coronal sections. Toluidine blue-stained semithin sectioning were prepared to determine the localization of neurons, and ultrathin sections were then cut and imaged using a Hitachi transmission electron microscope (TEM, Hitachi, Tokyo, Japan).

### Golgi staining

Golgi-Cox staining was performed using the FD Rapid Golgi Stain Kit. Fresh ischemic penumbra tissues were immersed in mixtures of equal parts of kit Solutions A and B and stored at RT for 2 weeks in the dark. Brain tissues were then transferred to solution C and kept at 4 °C for at least 48 h. All procedures were conducted in the dark. Samples were sliced into coronal sections with a thickness of 150 μm and stained according tothe manufacturer’s instructions.

### Sholl analysis

The morphology of dendritic complexity and the spine density were assessed using Sholl analysis. The number of intersections between apical and basal dendrites in 20 μm concentric circles around the centre of the cell soma was counted. Sholl analysis was used to assess the radial distribution of dendritic material and was conducted using the Sholl analysis plug-in (available at https://imagej.net/Sholl_Analysis) for ImageJ software (National Institutes of Health, Bethesda, MD, USA) to assess the number of bifurcations, the number of intersections, and the total length of the dendritic material contained in concentric circles.

### Measurement of spine density

The neuronal dendritic spine density was analysed within ischemic penumbra tissues. Layer V pyramidal cells were observed at 400× magnification, a length of dendrite was traced and the number of spines along the length was counted (to yield spines/10 μm), and no attempt was made to correct for hidden spines by overlying dendrites.

### Statistical analysis

SPSS 16.0 software was used for statistical analyses. Significant differences were analysed using one-way analysis of variance (ANOVA) followed by Dunnett's test (^#^*P* < 0.05, ^##^*P* < 0.01; ^*^P < 0.05, ^**^P < 0.01). All experimental data are presented as the mean ± standard deviation (SD), and *P* < 0.05 was considered to represent a statistically significant difference.
